# Trends and Age–Period–Cohort Effect on the Incidence of Early-Onset Colorectal Cancer (20–44 Years) from 1990 to 2021 in the United States

**DOI:** 10.3390/cancers16162883

**Published:** 2024-08-19

**Authors:** Wafa A. Aldhaleei, Michael B. Wallace, Akshaya Srikanth Bhagavathula

**Affiliations:** 1 Division of Gastroenterology and Hepatology, Mayo Clinic, Rochester, MN 55905, USA; aldhaleei.wafa@mayo.edu; 2 Division of Gastroenterology and Hepatology, Mayo Clinic, Jacksonville, FL 32224, USA; wallace.michael@mayo.edu; 3 Department of Public Health, North Dakota State University, Fargo, ND 58102, USA

**Keywords:** colorectal cancer, early-onset, incidence, age–period–cohort, global burden of diseases, epidemiology, public health, United States

## Abstract

**Simple Summary:**

The incidence of colorectal cancer in people under 50 years old is rapidly increasing in the United States. Our study aims to understand how often early-onset colorectal cancer occurs and what factors contribute to its rise. By analyzing data from 1990 to 2021, we found a significant increase in cancer cases, especially among women and those born after 1983. Our findings highlight the need for targeted prevention strategies and further research to uncover the reasons behind these trends. This research can help the medical community develop better screening and prevention methods to reduce the incidence of early-onset colorectal cancer.

**Abstract:**

The incidence of early-onset colorectal cancer (EO-CRC) in individuals under 50 years old is rapidly increasing in the United States. This study aims to evaluate EO-CRC incidence rates using data from the Global Burden of Disease Study (GBD) 2021, providing insights into trends from 1990 to 2021. We employed an age–period–cohort (APC) model analysis to estimate the effects of age, time period, and birth cohort on EO-CRC incidence. Our findings indicate that the number of EO-CRC cases rose from 6256 (95% UI: 6059–6456) in 1990 to 9311 (95% UI: 8859–9744) in 2021, a 49% increase from 1990 to 2021. The age-standardized incidence rate per 100,000 population increased by 34% during this period. The net drift in females (0.22%, 95% CI: 0.20–0.24) was slightly higher than in males (0.21%, 95% CI: 0.19–0.23) (*p* = 0.45). The APC analysis revealed that being over 25 years old, the period from 2005–2021, and being born after 1983 negatively impacted EO-CRC incidence rates, with a sharp rise after 2000 and a reduction among females from 2017 to 2021. Our study highlights the need for targeted prevention strategies and further research to understand these trends.

## 1. Introduction

The incidence of early-onset colorectal cancer (EO-CRC), defined as colorectal cancer diagnosed before age 50, has been increasing alarmingly in the United States (US) in recent decades [1,2]. The American Cancer Society projects that around 151,000 Americans will be diagnosed with colorectal cancer in 2024, with a rapid rise in cases among young adults [3]. Specifically, the incidence of EO-CRC has risen by 50% in the last 30 years, and by 2030, colorectal cancer (CRC) is predicted to be the leading cause of cancer death for the people under 50 in the US [4]. This escalating burden has been observed across all younger age groups from 20 to 49 years [5].

Several factors have been implicated in the rise of EO-CRC, including racial disparities, obesity, sedentary lifestyle, and environmental exposures [6,7]. African Americans have historically been at higher risk for EO-CRC compared to Caucasians [8], possibly due to socioeconomic factors and healthcare access. With the increase in the prevalence of risk factors in the younger population, the incidence of EO-CRC may continue to increase. 

Recent screening guidelines for the US population have lowered the recommended CRC screening age from 45 to 50 years for the average-risk population [9,10]. This change in the screening age threshold has consequently redefined the population considered unscreened for CRC. Previous studies examining the incidences and outcomes of EO-CRC did not account for this updated screening guideline [11,12,13]. An updated comprehensive analysis with a focus on the age group below 45 years is needed to provide a more accurate understanding of the evolving EO-CRC epidemiology.

Numerous studies have examined the epidemiological characteristics of EO-CRC at both the regional and national levels [14,15,16,17]. CRC is influenced by age-related factors; moreover, the epidemiology of CRC can be shaped by the specific time period and the birth cohort of the population. However, there is a notable gap in research examining how age, time period, and birth cohort influence the incidence of EO-CRC. Furthermore, few studies have utilized age–period–cohort (APC) models to evaluate these effects comprehensively. The Global Burden of Disease Study 2021 (GBD 2021) [18] evaluated over 370 diseases and injuries, providing comprehensive data to examine the epidemiological patterns and characteristics of EO-CRC. The age—period–cohort (APC) model is a powerful statistical method tool that can analyze trends in disease incidence and mortality by simultaneously accounting for three time-related factors: age, period, and cohort effects. This approach helps to separate these interconnected factors, offering a clear understanding of how each contributes to the observed trends in cancer incidence. By disentangling these intertwined effects, the APC model provides a clearer picture of how each factor contributes to the observed trends in cancer incidence [19].

In this study, we applied an APC model to analyze the incidence trends of EO-CRC in the US from 1990 to 2021, aiming to provide a comprehensive insight into the age, period, and cohort effects on EO-CRC, thereby informing targeted prevention strategies and future research directions.

## 2. Materials and Methods

### 2.1. Data Sources

We used aggregated national-level data provided by the Institute of Health Metrics and Evaluation (IHME) | Global Health Data Exchange (GHDx) to collect age-standardized incidence rates for EO-CRC in the US from 1990 to 2021. These data encompass age-standardized rates aggregated across the entire country, without individual or state-level breakdowns. The International Classification of Disease 10th Revision (ICD-10) codes used to define CRC (level 3) included C-180-18.9, C19, C19.0, C19.9, C2, C20, C20.0, C20.8, C20.9, C21, C21.0, C21.1, C21.2, C21.8, and C21.9. EO-CRC was defined in this study as the incidence of CRC before the age of 45 years. Age-standardized incidence rates per 100,000 population among males and females were used in our estimates.

These rates were standardized to the World Health Organization’s world standard population, allowing for adjustments to account for differences in age distribution within the population over time. This standardization is important for accurately comparing incidence rates across different demographic groups and over the study period.

### 2.2. Study Population

The study population included individuals aged 20 to 44 years diagnosed with colorectal cancer from 1990 to 2021 in the United States. The population was divided into five age groups: 20–24, 25–29, 30–34, 35–39, and 40–44 years.

### 2.3. Statistical Analysis

We used the APC model to analyze the incidence trends of EO-CRC. The APC model allows for the separation of the effects of age, period, and cohort that are often intertwined. The incidence of EO-CRC served as the dependent variable, while age, period, and birth cohort were considered as independent variables. The APC model uses statistical methods to analyze how age-specific time periods and birth years affect EO-CRC incidence rates over time. It helps to separate the influence of each factor on the incidence rates. 

The model assesses the impact of age, period, and cohort factors on the incidence rates, where the age effect reflects the difference in the EO-CRC incidence across various ages, the period effect shows the impact of different external factors during the study period (1990 to 2021) on EO-CRC incidence throughout all ages, and the cohort effect illustrates EO-CRC incidence changes caused by shared experiences or exposure to several risk factors across birth years.

The incidence ratio is a measure used to compare the incidence rates between different groups. It indicates how much more or less common EO-CRC is in one group compared to another. An incidence ratio greater than 1 suggests higher incidence, while a ratio less than 1 suggests lower incidence.

Ideally, the age and period intervals in the APC model should be equal. Because the age groups in the GBD 2021 are at five-year intervals, we displayed the incidence and population data in consecutive five-year periods (1990–1994, 1995–1999, 2000–2005, etc.), with 1990–1994 as the reference period. The population was divided into five age groups: 20–24, 25–29, 30–34, 35–39 and 40–44 years, with the medians of each interval as the reference age group.

The APC model produces two main results: net drift and local drift.

1. **Net drift** shows the overall annual increase in EO-CRC incidence rates across all birth cohorts.
2. **Local drift** indicates the annual increase in specific age groups, highlighting variations by age over time.

The cohorts are the result of participants’ period minus their age. The relative risk estimates and their 95% confidence intervals (CIs) were gathered using the Age–Period–Cohort web tool provided by the National Cancer Institute [19]. Other indicators include period rate ratios (RRs), birth cohort RRs, and fitted time trends.

### 2.4. Data Quality and Robustness

The estimates were generated using incidence and population data provided by the IHME and the National Center for Health Statistics. The IHME utilizes sophisticated modeling techniques to estimate disease incidence rates, incorporating data from various sources, including vital registration systems, cancer registries, and health surveys. These models adjust for potential biases and inconsistencies in the raw data, providing robust and reliable estimates of EO-CRC incidence rates.

### 2.5. Data Analysis and Visualization

Microsoft Excel 360 was used to collect the data. Figures were generated using the APC R software (version 4.2.2; R Foundation for Statistical Computing, Vienna, Austria). The statistical significance was set at a two-sided *p*-value < 0.05 using the APC’s Wald’s chi-square test.

## 3. Results

### 3.1. EO-CRC Incidence Trends

In the US, the burden of EO-CRC increased substantially from 1990 to 2021. The number of incident cases rose from 6256 (95% uncertainty interval [UI]: 6059–6456) in 1990 to 9311 (95% UI: 8859–9744) in 2021, representing a 49% increase (95% UI: 41–56%) during this period. Concurrently, the age-standardized incidence rate per 100,000 population increased significantly from 32.9 (95% UI: 31.8–33.9) in 1990 to 43.9 (95% UI: 41.8–46.0) in 2021, equating to a 34% (95% UI: 26–41%) overall increase in incidence rates from 1990 to 2021. The incidence rates of EO-CRC are generally higher in men compared to women across all age groups. More details are in Table 1.

**Figure 1:**
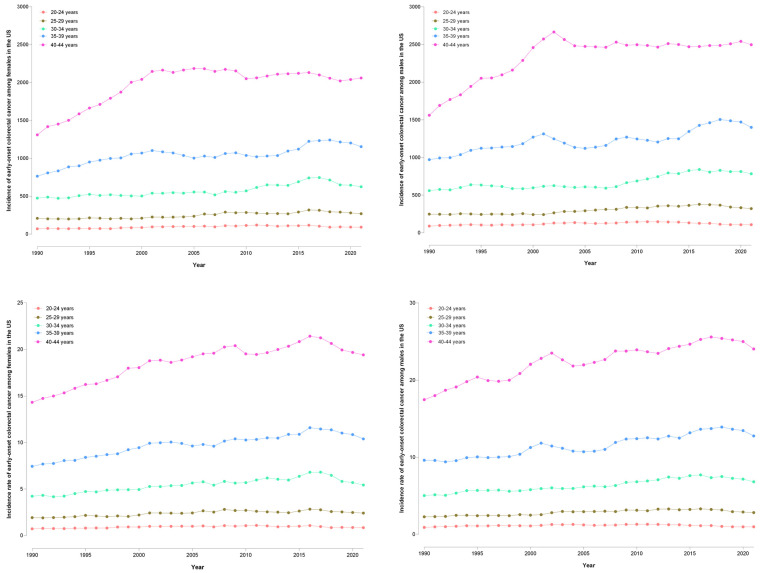
Trends in incident cases and incidence rate of early-onset colorectal cancer by age group and gender in the United States, 1990–2021 (females—left, and males—right).

In females, the 40–44 years age group saw an increase in incident EO-CRC cases from 1310 (95% UI: 1259–1365) in 1990 to 2060 (95% UI: 1942–2183) in 2021 (Figure 1). EO-CRC incidence in the 35–39 years age group rose from 764 to 1154 cases, while incidence in the 30–34 and 25–29 years groups increased from 500 and 300 cases to 700 and 500 cases, respectively. The 20–24 years group showed a slight rise from 70 to 92 cases. In males, the 40–44 years age group increased from 1560 to 2500 EO-CRC cases in 2021. The 35–39 years group rose from 1000 to 1400 cases, while the 30–34 and 25–29 years groups increased from 559 and 248 cases to 784 and 341 cases, respectively. The 20–24 years group saw a rise from 89 to 321 cases.

The mounting burden was observed across all younger age groups from 20 to 44 years. The largest percentage increase was observed among those aged 40–44 years, with a 59% escalation in incident cases overall (60% in males, 57% in females), coupled with a 37% increase in the incidence rate. Notably, even the youngest group, 20–24 years of age, exhibited a 26% surge in EO-CRC cases and a 13% higher incidence rate. Among individuals aged 35–39 years, cases increased by 47% overall, 51% in females, and 44% in males, while incidence rates increased by approximately 36–40% in both sexes. In the 30–34 years age group, a 36% increase in EO-CRC cases and a 32% elevation in incidence rate were noted across sexes. Although percentage increases tended to be higher among males across most age groups, females exhibited slightly greater increases in the 35–39 years age group.

### 3.2. Net and Local Drifts by Age Groups

**Figure 2:**
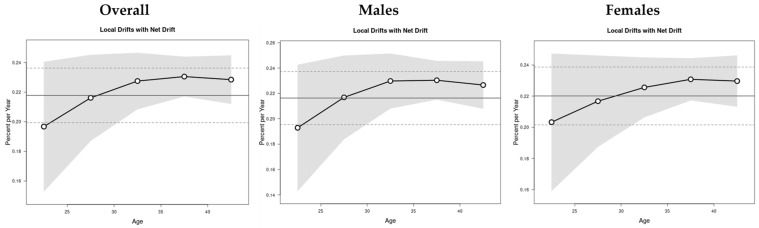
The net and local drifts of EO-CRC incidence from 1990 to 2021.

The net and local drifts were evaluated by age group, where net drift represents the overall annual percentage change across the study period, and the local drift represents the annual percentage change in the EO-CRC incidence rates relative to the net drift for each age group (Figure 2). The overall annual increase in EO-CRC incidence rates (net drift) was slightly higher in females (0.22% per year, 95% CI: 0.20 to 0.24) compared to males (0.21% per year, 95% CI: 0.19 to 0.23). However, this difference was not statistically significant (*p* = 0.45). The annual increase in specific age groups (local drift) was above average for both males and females, especially noticeable in individuals aged 22 years and older, indicating a worsening trend in EO-CRC incidence rates until around age 37.

### 3.3. APC Analysis

The figures illustrating changes in rates provide additional insights through the APC model analysis. This model isolates the effects of age, period, and cohort, offering a more nuanced understanding of EO-CRC incidence trends than simple incidence rate graphs.

**Figure 3:**
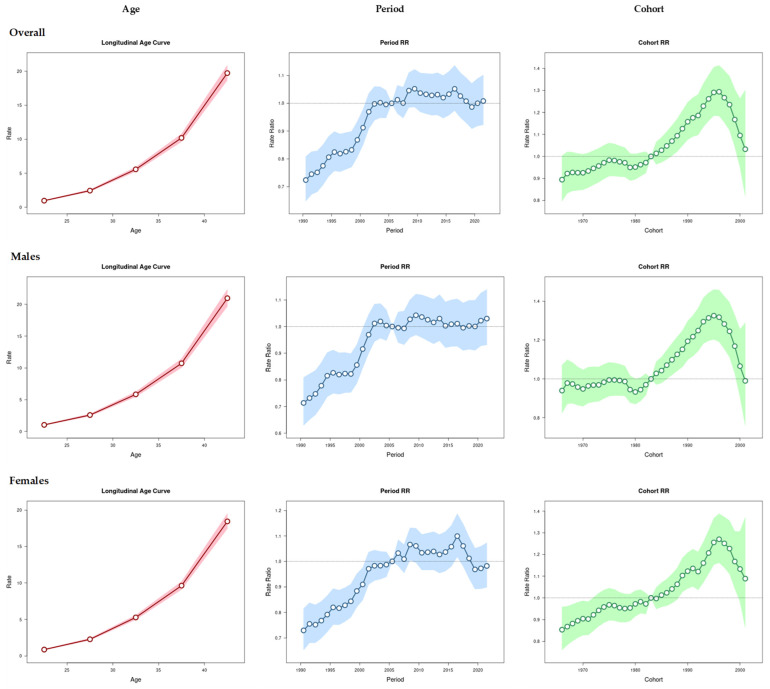
Age–period–cohort effects on EO-CRC incidence rates (males and females) from 1990 to 2021.

Figure 3 demonstrates the effect of age, period, and cohort on the EO-CRC incidence. Overall, the incidence of EO-CRC exhibits a rapid increase after the age of 25 years, irrespective of sex. However, the highest incidence is seen in males aged 42 years, around 21 per 100,000 population. From 2005, the incidence ratio of EO-CRC was greater than 1, indicating worsening EO-CRC incidence during these periods. However, the 2019–2021 period effect positively impacted the incidence rates in females. The cohort effect shows that individuals born in 1983 onwards had an incidence ratio greater than 1, with a bell-shaped curve indicating worsening incidence rates until 1996, followed by gradual reduction.

The APC analysis revealed that being over 25 years old, the period from 2005–2021, and being born after 1983 were important factors influencing EO-CRC incidence rates.

**Figure 4:**
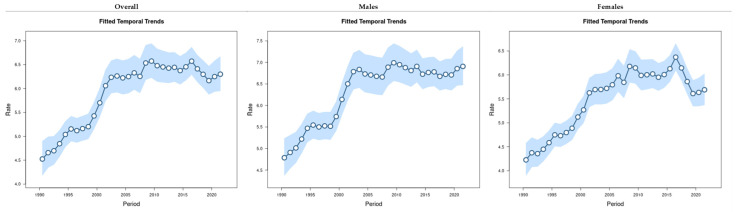
The overall age, period, and cohort effects on the EO-CRC incidence rates (per 100,000 population) by sex group from 1990 to 2021.

The fitted time trend represents the change in expected EO-CRC incidence over time for the reference age, adjusted for the cohort effects. The fitted time trend is increasing with a sharp increase after 2000, with an acute decrease in females from 2017 to 2021 (Figure 4).

## 4. Discussion

To the best of our knowledge, this is the first study to examine the long-term trends and APC effects of EO-CRC in the US from 1990 to 2021, using comprehensive data from the GBD 2021 study. Our study revealed a significant increase in EO-CRC incidence over the past three decades, with a 34% rise in age-standardized incidence rates from 32.9 per 100,000 in 1990 to 43.9 per 100,000 in 2021. The greatest increase was observed in individuals aged 40–44. The APC analysis highlighted critical factors influencing EO-CRC incidence, including being over 25 years old, the period from 2005–2021, and being born after 1983. These findings align with previous research, emphasizing the impact of lifestyle changes and genetic predispositions on EO-CRC trends.

The greatest increase was observed in individuals aged 40–44, with incident cases rising by 59% and incidence rates by 37%. This substantial rise highlights a growing public health concern that necessitates urgent attention. Local drift analysis indicated an increase in EO-CRC incidence among those aged 22 and above, peaking at age 42. The cohort effect showed that individuals born after 1983 were at elevated risk, with the incidence ratio peaking around 1996 before declining. This suggests that younger cohorts may be experiencing different or additional risk factors compared to older cohorts.

Our findings are consistent with previous research showing a significant global rise in EO-CRC incidence. Patel et al. (2021) emphasized the rising burden in individuals under 50, linking EO-CRC trends to lifestyle changes and genetic predispositions [20]. Their recommendations led to updated guidelines for colorectal cancer screening, advocating for earlier screening starting at age 45 due to the high prevalence of neoplasia in the young population. Similarly, a global systematic review also documented an increase in young-onset CRC across multiple countries, particularly in North America and Europe [21,22,23].

Multiple studies have emphasized that the increased incidence in EO-CRC is primarily attributed to shifts in dietary patterns and increasingly sedentary lifestyle, which exacerbate CRC risk [20,24,25,26,27,28,29,30]. A case-control study by Low et al. identified obesity, diabetes, and smoking as prominent risk factors for EO-CRC among US veterans aged 18–49 [24]. Additionally, the study highlighted the complex interplay of diet and metabolic health, identifying regular alcohol consumption, hypertension, higher BMI, and increased fish consumption as key contributors to EO-CRC in a Chinese population [25]. While fish consumption was initially identified as a potential risk factor, this finding is unusual. Most CRC epidemiological studies indicate that fish is either neutral or protective, except in specific regions like northern Iceland where fish contamination is an issue. Furthermore, sedentary behavior, unhealthy diet, and family history, increased metabolic risk factors such as hypertension, dyslipidemia, and type 2 diabetes significantly raise the likelihood of developing colorectal adenocarcinoma [26,27,28,29,30].

Findings from our APC analysis highlighted the importance of distinguishing between age, period, and cohort effects in understanding EO-CRC trends. The rapid increase in incidence after age 25, irrespective of sex, suggests that interventions targeting young adults could be crucial. Moreover, the period effect from 2005 onwards, which showed a worsening trend, indicates that recent environmental or lifestyle changes are likely contributors to the rising incidence. This aligns with the observed increase in incidence rates among individuals born after 1983, peaking around 1996, which could reflect generational shifts in risk factors.

Although our study did not assess EO-CRC by cancer location due to data constraints, it is essential to note that a combination of sigmoidoscopy and fecal immunochemical test (FIT) is most appropriate for distal/rectal cancer screening, while colonoscopy and/or FIT are adequate for proximal colon screening. Future studies should aim to include cancer location to provide more specific screening recommendations.

Considering the risk factors associated with EO-CRC, they underscore the importance of adopting a multifaceted approach to prevention. Dietary guidelines should emphasize a balanced and nutrient-rich diet, as a well-rounded nutritional intake plays a critical role in reducing EO-CRC risk. Coupled with the promotion of regular physical activity, these lifestyle modifications can significantly mitigate the risk of this condition. Furthermore, targeted health screening, particularly among high-risk cohorts born between 1983 and 2016, is crucial for early detection and timely treatment, which can further aid in the prevention and management of EO-CRC.

The escalating burden of EO-CRC necessitates immediate action from healthcare policymakers and clinicians. While current screening guidelines recommend colorectal cancer screening from age 45 onwards for the average-risk populations, evidence suggests that even younger individuals may be at significant risk, particularly those with a family history or known genetic predispositions. Therefore, a comprehensive understanding of lifestyle factors impacting EO-CRC risk across different demographics and age groups is crucial. Analyzing genetic markers can help identify specific high-risk populations that would benefit from targeted screening and preventive measures. Additionally, evaluating the effectiveness of current screening guidelines and considering lowering the recommended screening age for certain high-risk groups under 45 is imperative. Furthermore, investigating socioeconomic disparities in healthcare access can shed light on the reasons behind delayed diagnoses and worse outcomes, enabling the development of targeted interventions to address these inequalities. By conducting multifaceted research, tailored strategies for prevention, early detection, and improved outcomes can be developed to combat the rising burden of EO-CRC.

### Strengths and Limitations

A significant strength of this study is its use of the comprehensive GBD 2021 dataset, providing long-term data across 31 years for robust analysis. The APC model allowed a detailed exploration of age, period, and cohort effects on EO-CRC incidence. However, the study is limited by its reliance on aggregated national data, which may obscure regional disparities in EO-CRC trends. The GHDx dataset did not provide information on cancer location (proximal colon, distal colon, or rectal cancers). Additionally, lifestyle risk factors and genetic predispositions were not directly analyzed due to data constraints. We acknowledge the importance of analyzing ethnicity in CRC incidence studies. While our current study does not include this analysis due to data limitations, we plan to address this significant aspect in future research. Furthermore, reliance on public data might have introduced inaccuracies or inconsistencies in cancer classification.

## 5. Conclusions

Our study delivers important insights into the increasing incidence of EO-CRC in the US over the past three decades. Our findings reveal a consistent and substantial rise in incidence rates, particularly among individuals aged 40–44. The APC analysis underscores the complex interplay between age, period, and cohort effects. Our research strengthens the call for refining early detection strategies, increasing public awareness, and expanding studies into the genetic and lifestyle factors contributing to the growing EO-CRC burden. Addressing these issues through targeted research and public health interventions will be crucial in mitigating the future impact of this disease.

## Figures and Tables

**Table 1:** Early-onset colorectal cancer incidence and percentage change from 1990–2021.

	Incident Cases (95% UI)		Percentage Change (95% UI)	Incidence Rate per 100,000 (95% UI)		Percentage Change (95% UI)
**Overall**	1990	2021	1990–2021	1990	2021	1990–2021
20–24	159 (154–164)	200 (190–209)	0.26 (0.18–0.33)	0.81 (0.78–0.84)	0.92 (0.87–0.96)	0.13 (0.06–0.19)
25–29	457 (475–440)	591 (557–620)	0.29 (0.20–0.38)	2.10 (2.02–2.19)	2.62 (2.48–2.75)	0.25 (0.16–0.33)
30–34	1034 (1001–1068)	1409 (1336–1477)	0.36 (0.28–0.45)	4.63 (4.48–4.78)	6.13 (5.81–6.42)	0.32 (0.24–0.41)
35–39	1736 (1684–1787)	2554 (2431–2664)	0.47 (0.39–0.55)	8.52 (8.27–8.77)	11.57 (11.01–12.07)	0.36 (0.28–0.43
40–44	2870 (2789–2954)	4557 (4354–4774)	0.59 (0.50–0.67)	15.88 (15.43–16.35)	21.69 (20.73–22.72)	0.37 (0.29–0.44)
**Females**						
20–24	70 (67–73)	92 (87–97)	0.31 (0.21–0.41)	0.73 (0.70–0.76)	0.86 (0.81–0.91)	0.17 (0.09–0.27)
25–29	209 (200–219)	270 (251–287)	0.29 (0.18–0.39)	1.93 (1.85–2.02)	2.42 (2.25–2.57)	0.25 (0.15–0.36)
30–34	475 (451–497)	625 (582–667)	0.32 (0.20–0.45)	4.24 (4.03–4.44)	5.44 (5.07–5.81)	0.28 (0.17–0.41)
35–39	764 (735–795)	1154 (1080–1235)	0.51 (0.38–0.63)	7.45 (7.16–7.75)	10.40 (9.73–11.12)	0.40 (0.28–0.51)
40–44	1310 (1259–1365)	2060 (1942–2183)	0.57 (0.46–0.68)	14.33 (13.77–14.93)	19.41 (18.30–20.56)	0.35 (0.26–0.44)
**Male**						
20–24	89 (85–92)	108 (102–114)	0.22 (0.13–0.31)	0.89 (0.85–0.92)	0.97 (1.03–0.92)	0.09 (0.01–0.17)
25–29	248 (237–259)	321 (102–114)	0.30 (0.20–0.41)	2.27 (2.17–2.37)	2.82 (2.63–3.00)	0.24 (0.15–0.35)
30–34	559 (537–583)	784 (300–341)	0.40 (0.30–0.51)	5.02 (4.82–5.23)	6.81 (6.41–7.19)	0.36 (0.26–0.46)
35–39	971 (932–1010)	1400 (1317–1482)	0.44 (0.34–0.54)	9.61 (9.22–9.99)	12.76 (12.00–13.51)	0.33 (0.24–0.42)
40–44	1560 (1506–1617)	2497 (2351–2636)	0.60 (0.50–0.71)	17.47 (16.86–18.10)	24.02 (22.62–25.35)	0.38 (0.29–0.47)

UI: uncertainty interval.

## Data Availability

The data related to this research is available from https://ghdx.healthdata.org/gbd-2021.

## References

[1] Rex D.K., Boland C.R., Dominitz J.A., Giardiello F.M., Johnson D.A., Kaltenbach T., Levin T.R., Lieberman D., Robertson D.J. (2017). Colorectal cancer screening: Recommendations for physicians and patients from the US Multi-Society Task Force on Colorectal Cancer. Gastroenterology.

[2] Patel S.G., Karlitz J.J., Yen T., Lieu C.H., Boland C.R. (2022). The rising tide of early-onset colorectal cancer: A comprehensive review of epidemiology, clinical features, biology, risk factors, prevention, and early detection. Lancet Gastroenterol. Hepatol..

[3] Siegel R.L., Giaquinto A.N., Jemal A. (2024). Cancer statistics, 2024. CA Cancer J. Clin..

[4] Bailey C.E., Hu C.Y., You Y.N., Bednarski B.K., Rodriguez-Bigas M.A., Skibber J.M., Cantor S.B., Chang G.J. (2015). Increasing disparities in the age-related incidences of colon and rectal cancers in the United States, 1975–2010. JAMA Surg..

[5] Montminy E.M., Zhou M., Maniscalco L., Penrose H., Yen T., Patel S.G., Wu X.C., Karlitz J.J. (2021). Trends in the Incidence of Early-Onset Colorectal Adenocarcinoma Among Black and White US Residents Aged 40 to 49 Years, 2000–2017. JAMA Netw. Open.

[6] Wu C.W., Lui R.N. (2022). Early-onset colorectal cancer: Current insights and future directions. World J. Gastrointest. Oncol..

[7] Ullah F., Pillai A.S.B., Omar N., Dima D., Harichand S. (2023). Early-Onset Colorectal Cancer: Current Insights. Cancers.

[8] Carethers J.M. (2015). Screening for colorectal cancer in African Americans: Determinants and rationale for an earlier age to commence screening. Dig. Dis. Sci..

[9] Wolf A.M.D., Fontham E.T.H., Church T.R., Flowers C.R., Guerra C.E., LaMonte S.J., Etzioni R., McKenna M.T., Oeffinger K.C., Shih Y.T. (2018). Colorectal cancer screening for average-risk adults: 2018 guideline update from the American Cancer Society. CA Cancer J. Clin..

[10] Davidson K.W., Barry M.J., Mangione C.M., Cabana M., Caughey A.S.B.., Davis E.M., Donahue K.E., Doubeni C.A., Krist A.H., US Preventive Services Task Force (2021). Screening for Colorectal Cancer: US Preventive Services Task Force Recommendation Statement. JAMA.

[11] Montminy E.M., Zhou M., Maniscalco L., Heda R., Kim M.K., Patel S.G., Wu X.C., Itzkowitz S.H., Karlitz J.J. (2022). Shifts in the Proportion of Distant Stage Early-Onset Colorectal Adenocarcinoma in the United States. Cancer Epidemiol. Biomark. Prev..

[12] Gabriel E., Attwood K., Al-Sukhni E., Erwin D., Boland P., Nurkin S. (2018). Age-related rates of colorectal cancer and the factors associated with overall survival. J. Gastrointest. Oncol..

[13] Gabriel E., Ostapoff K., Attwood K., Al-Sukhni E., Boland P., Nurkin S. (2017). Disparities in the Age-Related Rates of Colorectal Cancer in the United States. Am. Surg..

[14] Shah R.R., Millien V.O., da Costa W.L., Oluyomi A.O., Gould Suarez M., Thrift A.P. (2022). Trends in the incidence of early-onset colorectal cancer in all 50 United States from 2001 through 2017. Cancer.

[15] Abualkhair W.H., Zhou M., Ahnen D., Yu Q., Wu X.C., Karlitz J.J. (2020). Trends in incidence of early-onset colorectal cancer in the United States among those approaching screening age. JAMA Netw. Open.

[16] Cercek A., Chatila W.K., Yaeger R., Walch H., Fernandes G.D.S., Krishnan A., Palmaira L., Maio A., Kemel Y., Srinivasan P. (2021). A Comprehensive Comparison of Early-Onset and Average-Onset Colorectal Cancers. J. Natl. Cancer Inst..

[17] Akimoto N., Ugai T., Zhong R., Hamada T., Fujiyoshi K., Giannakis M., Wu K., Cao Y., Ng K., Ogino S. (2021). Rising incidence of early-onset colorectal cancer—A call to action. Nat. Rev. Clin. Oncol..

[18] GBD 2021 Diseases and Injuries Collaborators (2024). Global incidence, prevalence, years lived with disability (YLDs), disa-bility-adjusted life-years (DALYs), and healthy life expectancy (HALE) for 371 diseases and injuries in 204 countries and territories and 811 subnational locations, 1990–2021: A systematic analysis for the Global Burden of Disease Study 2021. Lancet..

[19] Rosenberg P.S., Check D.P., Anderson W.F. (2014). A web tool for age-period-cohort analysis of cancer incidence and mortality rates. Cancer Epidemiol. Biomark. Prev..

[20] Patel S.G., May F.P., Anderson J.C., Burke C.A., Dominitz J.A., Gross S.A., Jacobson B.C., Shaukat A., Robertson D.J. (2022). Updates on age to start and stop colorectal cancer screening: Recommendations from the U.S. Multi-Society Task Force on Colorectal Cancer. Gastrointest. Endosc..

[21] Saad El Din K., Loree J.M., Sayre E.C., Gill S., Brown C.J., Dau H., De Vera M.A. (2020). Trends in the epidemiology of young-onset colorectal cancer: A worldwide systematic review. BMC Cancer.

[22] Voigtländer S., Hakimhashemi A., Grundmann N., Rees F., Meyer M., Algül H., Müller-Nordhorn J. (2022). Trends of colorectal cancer incidence according to age, anatomic site, and histological subgroup in Bavaria: A registry-based study. Front. Oncol..

[23] Schell D., Ullah S., Brooke-Smith M.E., Hollington P., Yeow M., Karapetis C.S., Watson D.I., Pandol S.J., Roberts C.T., Barreto S.G. (2022). Gastrointestinal Adenocarcinoma Incidence and Survival Trends in South Australia, 1990–2017. Cancers.

[24] Low E.E., Demb J., Liu L., Earles A., Bustamante R., Williams C.D., Provenzale D., Kaltenbach T., Gawron A.J., Martinez M.E. (2020). Risk Factors for Early-Onset Colorectal Cancer. Gastroenterology.

[25] Pan Z., Huang J., Huang M., Yao Z., Huang J., Chen J., Yu X., Wang R. (2023). Risk factors for early-onset colorectal cancer: A large-scale Chinese cohort study. J. Natl. Cancer Cent..

[26] O’Sullivan D.E., Sutherland R.L., Town S., Chow K., Fan J., Forbes N., Heitman S.J., Hilsden R.J., Brenner D.R. (2022). Risk Factors for Early-Onset Colorectal Cancer: A Systematic Review and Meta-analysis. Clin. Gastroenterol. Hepatol..

[27] Hua H., Jiang Q., Sun P., Xu X. (2023). Risk factors for early-onset colorectal cancer: Systematic review and meta-analysis. Front. Oncol..

[28] Li Q., Weitz J., Li C., Schardey J., Weiss L., Wirth U., Zimmermann P., Bazhin A.V., Werner J., Kühn F. (2023). Smoking as a risk factor for colorectal neoplasms in young individuals? A systematic meta-analysis. Int. J. Color. Dis..

[29] Khoa Ta H.D., Nguyen N.N., Ho D.K.N., Nguyen H.D., Ni Y.C., Yee K.X., Pan S.R., Nguyen H.S., Thai Hoang Phuoc T., Chen M.-J. (2023). Association of diabetes mellitus with early-onset colorectal cancer: A systematic review and meta-analysis of 19 studies including 10 million individuals and 30,000 events. Diabetes Metab. Syndr..

[30] Schumacher A.J., Chen Q., Attaluri V., McLemore E.C., Chao C.R. (2021). Metabolic Risk Factors Associated with Early-Onset Colorectal Adenocarcinoma: A Case-Control Study at Kaiser Permanente Southern California. Cancer Epidemiol. Biomark. Prev..

